# Implementing a combined Delphi and Focus Group qualitative methodology in Nexus research designs—The case of the WEFE Nexus in Apokoronas, Crete

**DOI:** 10.1371/journal.pone.0271443

**Published:** 2022-07-14

**Authors:** Carolin Canessa, Andreas Vavvos, Sofia Triliva, Iosif Kafkalas, Maria Vrachioli, Johannes Sauer

**Affiliations:** 1 Department of Agricultural Production and Resource Economics, Technical University of Munich, Freising, Germany; 2 Department of Psychology, University of Crete, Rethymnon, Crete, Greece; 3 Department of Economics, University of Crete, Rethymnon, Crete, Greece; Soil and Water Resources Institute ELGO-DIMITRA, GREECE

## Abstract

In recent years, researchers and policymakers have emphasised the importance of understanding the complex relationships between Water, Energy, Food and Ecosystems (WEFE). The primary reason for capturing these complexities is to understand how decisions made in the water, food and energy sectors can affect one another. Crucially, biodiversity and ecosystem services (E) play a mediating role in these relationships by making material and non-material contributions to all other sectors (W, E, F). The Nexus approach has been widely used for capturing these interdependencies and identifying opportunities for increasing efficiency, reducing trade-offs and building synergies for sustainable resource use across the WEFE nodes. One challenge in using this framework is the need to harmonise the technical and managerial dimensions of the WEFE interlinkages with the perceptions and priorities of local populations directly involved in the use and management of resources. This paper presents a methodological framework that seeks to integrate the perspectives of experts, practitioners and local stakeholders on the WEFE Nexus through the combined application of the Delphi and Focus Group methods. In this paper, the municipality of Apokoronas in Crete, Greece has served as the case in point. The combined framework allowed us to explore the Nexus understanding at the local level and was instrumental in the identification of initiatives for more integrated resource management. The triangulation of results captured the differences in priorities between practitioners and the local community at large, but also, more specifically, it pointed to discrepancies within groups and across WEFE sectors. The outcomes of this paper demonstrate that awareness and learning play a central role in Nexus actions to overcome conflicts and perceived inequalities, and to internalise solutions. The inclusion of the ecosystems node in the traditional WEF Nexus encouraged participants to contemplate the pivotal role of ecosystems in supporting the rest of the WEF sectors.

## 1 Introduction

In recent years, researchers and policymakers have emphasised the importance of understanding the complex relationships between water, energy, food and ecosystems [1–4]. The interdependencies between these sectors are commonly referred to as the Water-Energy-Food-Ecosystems (WEFE) Nexus. The implication behind this concept is that in a world of rising global population and increasing pressure on resources, any strategy that is not nodal—focuses on one sector at a time without taking into account its impact on others—can have serious unintended consequences and result in oversimplified and narrow theoretical approaches that ignore the notion of interconnectivity [5, 6]. Against this frame of reference, accounting for the interlinkages of WEFE nodes through a Nexus approach has become a priority in the global development agenda [7]. The Nexus approach is a means to identify opportunities for increasing efficiency, reducing trade-offs and building synergies for sustainable resource use across the nodes [8].

Despite the fact that the scope of the approach is clear [9], the implementation of the framework can still be challenging in the face of the complex (spatial, temporal, institutional and jurisdictional) interlinkages across the various sectors [10, 11]. One of the thorniest problems to plague the operationalisation of the WEFE concept pertains to grasping fully all the biophysical and socio-economic links within and across nodes. This is possibly the main reason why the ecosystems node is frequently omitted by the majority of Nexus studies [12]. To date, most published research on the WEFE Nexus only partially includes a biophysical and economic assessment of ecosystem services [2]. Moreover, the Nexus framework has been criticised as a construct that obscures the power inequalities inherent in the use and management of natural resources, by focusing solely on technical, technological and managerial solutions. Additionally, the concept is perceived as poorly connected with local visions of sustainable and just futures, as well as issues of environmental justice and poverty [13, 14].

Since the introduction of the concept in 2011 [8], researchers and policymakers have made significant efforts to explore the Nexus from a variety of perspectives [15]. Over the past decade, nearly three-quarters of all studies on the Nexus have been quantitative in nature, favouring its use as a tool to assess the biophysical resource flows between nodes [16]. At the same time, however, a relatively small body of literature has studied the concept under a qualitative lens [15], using a wide range of methods and combinations thereof, such as fuzzy cognitive mapping [17], stakeholder analysis [18, 19], expert interviews [18, 20] and workshops [9, 17, 19]. Due to the high level of technical expertise required, these studies have often targeted communities of experts and policymakers, and seldom addressed wider groups of stakeholders or citizens. This is also the case in the studies by Smajgl et al. [21] and Foran et al. [22], who used the Delphi method to investigate the implications of large-scale development investments on the Water-Energy-Food (WEF) Nexus in the Mekong basin. As a result, stakeholder engagement within the Nexus research agenda is still limited, despite widespread recognition of the potential value of interdisciplinary research [23]. Several authors agree that participatory processes, convergence thinking, and interdisciplinary approaches can bring new perspectives into the discussion, by supporting knowledge-sharing and informed decision-making [9, 24].

A critical reconsideration of the Nexus concept is of particular relevance for the Mediterranean region. The adoption of an integrated perspective in resource management has become a priority, due to the growing demand for resources, increased water scarcity, climate change, the degradation of ecosystem services, and water pollution in the region [25]. In the face of these developments, scientists and policymakers have emphasised the need to promote food and water security through integrated resource management and inclusive development, which recognise the role of different stakeholders in society [26]. Several studies have investigated the management of two or three WEFE resources in the Mediterranean, using qualitative methods [18, 27]. Karabulut et al. [28] and Laspidou et al. [29] have proposed Nexus approaches to quantify the interlinkages among different sectors, including climate, land and environment. Karabulut et al. [30] solicited expert opinions to assess the cross-sectoral impacts of different European policies on the WEFE Nexus, while Malagó et al. [31] proposed an analytical framework to identify and balance the strengths and weaknesses of WEFE Nexus practices in the Mediterranean context. To the best of our knowledge, however, no study to date has used either the Delphi or the Focus Group techniques to investigate stakeholder understanding of the WEFE Nexus in the Mediterranean, in a context where equal importance is placed to all four nodes of the Nexus.

A compelling case can be made for adopting a more comprehensive qualitative approach to the study of the WEFE Nexus, as there is documented need to understand the diverse interactions between WEFE nodes and people in the Mediterranean region [29]. Within this context, this paper explores local stakeholders’ understanding of the WEFE Nexus by applying the Focus Group and Delphi methods to the area of Apokoronas in Crete, Greece. The Delphi (DS) and Focus Group (FG) techniques are used in the same study to investigate how local experts and stakeholders, respectively, view the WEFE Nexus and its concomitant resource management integration strategies. While Delphi attempts to capture the overall existing pressures over the Nexus and is predicated on expert opinions, the FGs investigate the "context-embedded" perspectives of various groups of local stakeholders regarding the interplay between resources, policies and sustainable development. The results of the study were compared by triangulating outcomes from both methodologies [32]. The following research questions guided the analysis: i) How do diverse stakeholders understand and frame the interconnections and interdependencies across the WEFE sectors? ii) What types of solutions would the stakeholders put in place to improve WEFE integrated resource management? iii) What are the prospects of applying qualitative methodologies in Nexus research designs?

The analysis is structured as follows. Section 2 introduces the case study area and the methodology. It presents the data collection process and the demographic characteristics of the participants, and discusses the appropriateness of the methods used. Section 3 contains an independent presentation of the results of the Delphi surveys and the Focus Groups interviews in light of the research questions. In Section 4, the results from both methodologies are triangulated and policy implications are discussed. Finally, future areas of investigation are proposed.

## 2 Materials and methods

### 2.1 Study area

**Fig 1:**
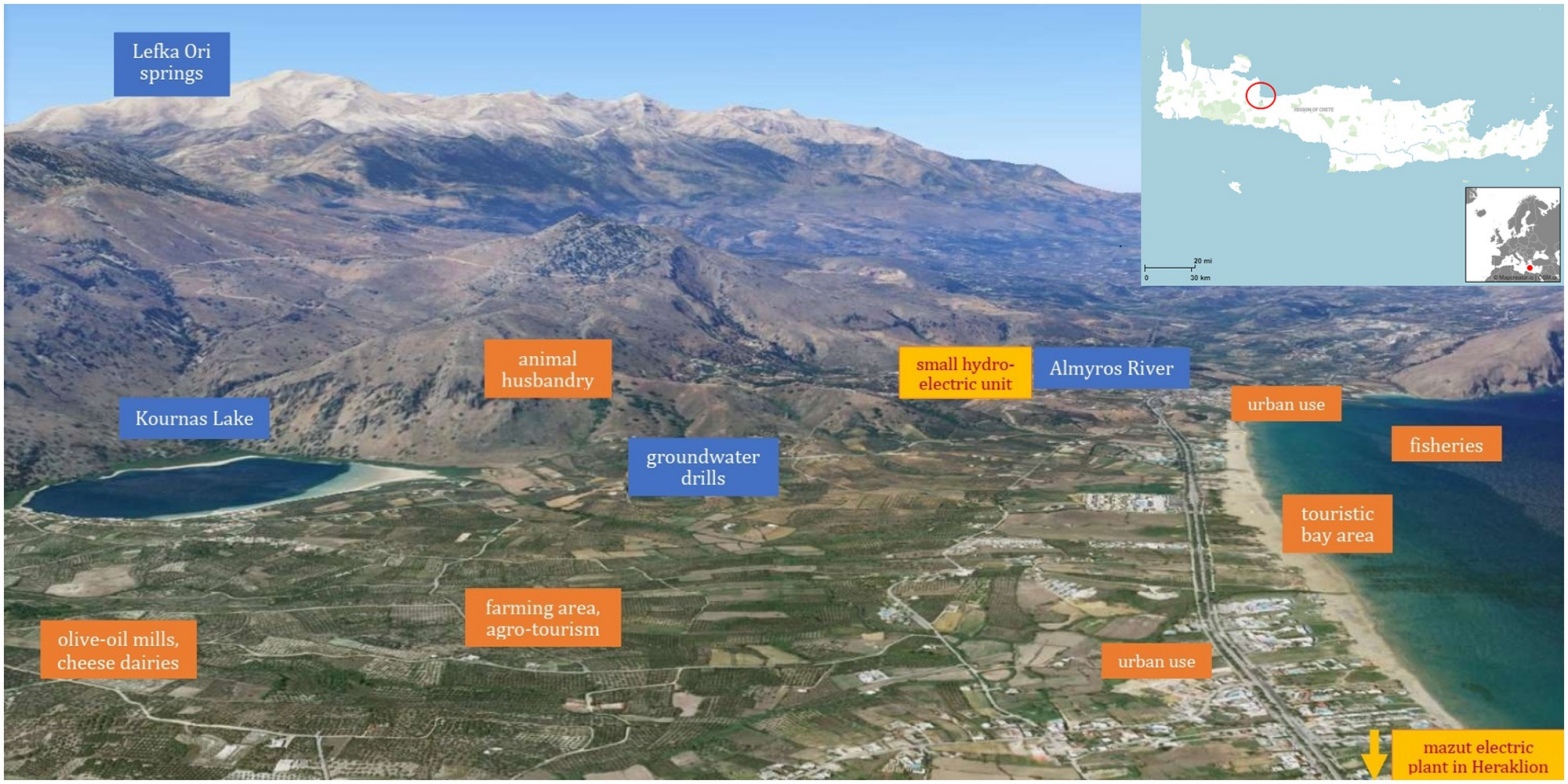
Case study area location and WEFE Nexus components.

Apokoronas is a district of 15,289 inhabitants located in the north-eastern corner of the Chania prefecture of Crete, at the foot of the White Mountains (Fig 1) [33]. According to Köppen-Geiger climate classification system, the climate in the study area is warm and temperate. The average maximum temperature is 23.6 °C, the average minimum temperature is 11.3°C and the annual precipitation is around 900 mm. The economy in the study area relies on agriculture, including many small-scale dairy and olive-oil facilities, and tourism. The study area aims to reflect the impact of both physical and anthropogenic drivers affecting the WEFE Nexus. The physical drivers are associated mainly with the impacts of climate change, while the anthropogenic drivers reflect demographic changes, the transition from traditional crops to new water-intensive ones, and the continuous touristic development along with higher hosting standards.

#### 2.1.1 Water in Apokoronas

The island of Crete is self-sufficient in terms of water supply, but still commonly suffers from water scarcity events. These are mainly due to local climatic conditions and disparities between water availability and demand [34]. The municipality of Apokoronas registers the highest precipitation and hosts the only natural freshwater lake on the island; yet, it experiences increased conflict over resource allocation. Lake Kournas and its freshwater system belong to the Natura 2000 Network owing to its rich ecosystem and soil [35, 36]. The lake of Kournas is the only natural freshwater lake in Crete. Its hydrological system supplies nearby villages and supports a vast peripheral area of arable land, which, especially around the Almyros estuary, is particularly fertile. In the past 30 years, there has been an expansion in service industries on the island (mainly tourism-related) at the expense of farming areas. The model of rapid tourism development, and, in some cases over-tourism, in Crete is subject to increased criticism for its negative impact on natural resources and the environment [37].

#### 2.1.2 Energy in Apokoronas

From the energy perspective, Crete’s electrical grid was recently connected to mainland Greece’s power supply. Whilst electricity production on the island is partly based on thermal power units that operate on fossil fuels, the connection to the mainland electrical grid created the conditions for increasing renewable energy production for the years to come. Many inhabitants use small energy generating turbines for pumping water and a biofuel produced from olive kernels for heating purposes. A small hydroelectric unit operates in Almyros river in Georgioupoli, where water from the natural lake and the Lefka Ori mountain springs is used as a source of electricity.

#### 2.1.3 Food in Apokoronas

Agriculture remains a very important and dynamic sector in the area of Apokoronas with farmers cultivating traditional crops, such as olives and citrus fruits, or newly introduced, water-intensive crops, such as avocados. Other key agricultural sub-sectors include livestock, agro-forestry, and fishing. Farmers engage in a range of interdependent gathering, production and post-harvesting processes and their livelihoods encompass various processing-packaging sector activities. Similarly, people who are employed or own family-run businesses also farm their own land.

#### 2.1.4 Ecosystems in Apokoronas

The entire freshwater system around Kournas lake, including the marshes, the Almyros stream and estuary in Georgioupolis, are one of the most important in the east Mediterranean region [34]. The area is a relevant site for breeding, migrant and wintering waterbirds. Species of concern include Egrettagarzetta and Aythya Nyroca. The local ecosystem supports the essential provision, regulation and cultural services, which are pressured by complex, transactional and anthropogenic drivers of change such as tourism, agriculture, inappropriate water and environmental management [38].

### 2.2 Delphi study methodology

The Delphi technique refers to a structured, anonymous and iterative round of surveys aimed at eliciting opinions from a panel of experts, who are asked to reach a consensus on critical issues [39]. The method is used to structure a group communication process, whilst avoiding confrontation among the participants [40]. Different variations of Delphi studies exist, depending on their objectives [41–43]. In this study, a modified argument-policy Delphi was adopted, which was simultaneously investigating expert understanding of the WEFE Nexus in Apokoronas and opportunities for its integrated management [44]. The technique was used for exploratory rather than confirmatory purposes. As a result, the consensus measurement, typical of the Delphi technique, was not its main goal but rather the instrument for data analysis.

**Fig 2:**
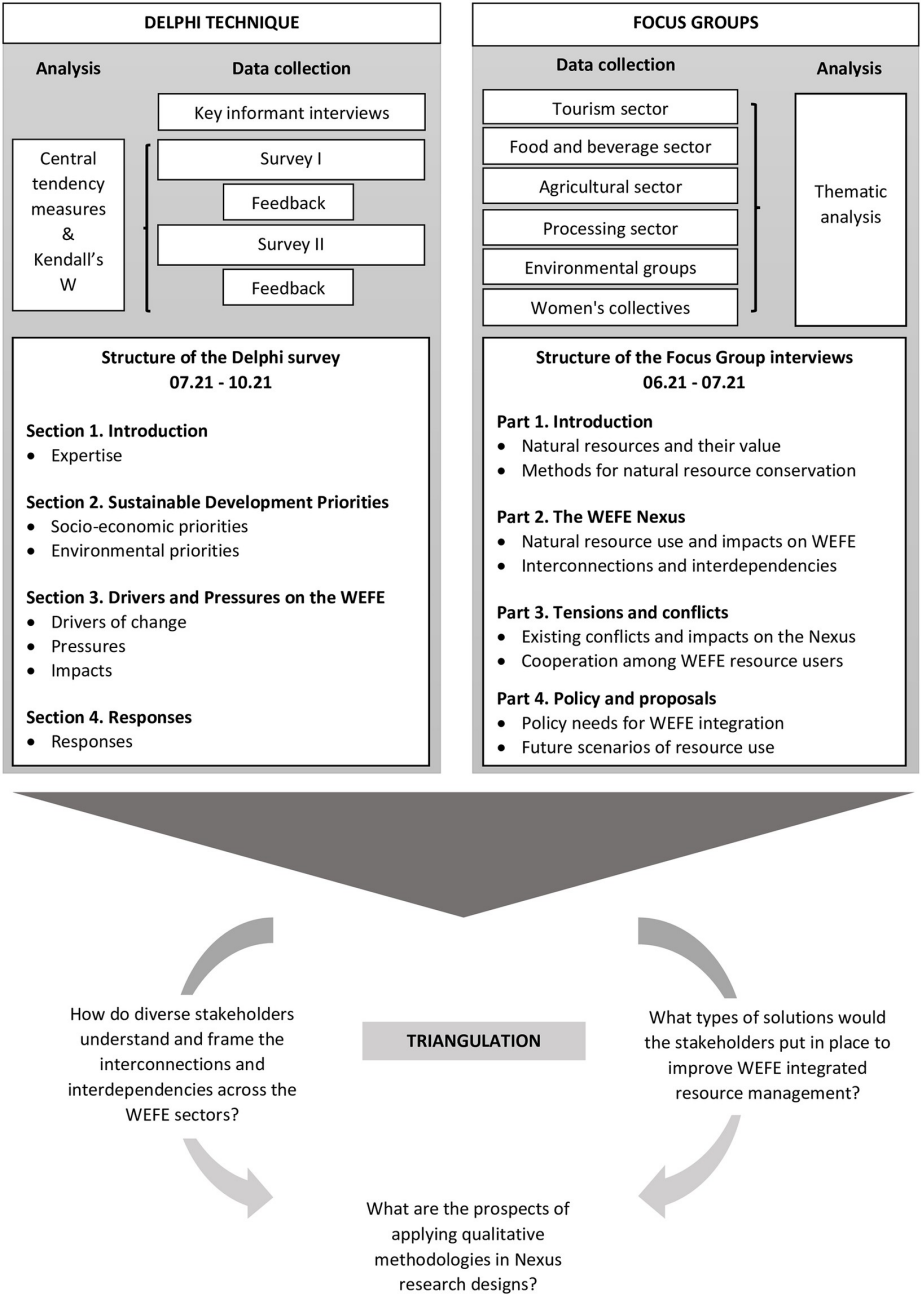
Methodological flow chart of the study.

Following the Delphi criteria of iteration, anonymity and controlled feedback, two rounds of quantitative surveys, preceded by key informant interviews, were developed [45]. The use of a qualitative round is widely accepted and strongly encouraged [46]. In terms of structuring the questionnaire, a general-to-specific approach was used to allow experts to evaluate the questions in increasing depth. The questionnaires included a combination of closed- and open-ended questions designed based on the outcomes of the key informant interviews. As outlined in Fig 2, the survey consists of four sections that cover the expertise background of the interviewees, the sustainable development priorities for the area, the drivers and pressures on the WEFE Nexus in the area, and the possible responses to increase WEFE Nexus integrated management in Apokoronas. A simplified Drivers, Pressures, State, Impact and Response (DPSIR) logic was used to guide the survey design and the interpretation of the results [47]. The survey concentrated mainly on the Drivers-Pressures-Impact and Responses on the WEFE Nexus, and avoided the details on the “state of the environment” to reduce the complexity of the task for the experts. This approach was previously used by Benitez-Capistros et al. [48] in combination with the Delphi to investigate proper conservation management strategies in the Galapagos Islands. In the context of this research, the simplified DPRI was found to be the most appropriate approach to explore the relations between socio-ecological systems and the impacts on the four Nexus nodes.

In the second round of the survey, the experts were introduced to the results of the first round and were asked to revise their answers. Only those questions where no consensus was achieved in the first round were included in the second. For the data analysis of each round, conventional central tendency measures were calculated [49]. Moreover, different measures of consensus to measure polarisation among individuals were also used: i) the Kendall’s W coefficient of concordance (KW) to rank questions (KW: 0–0.49 for non-consensus, KW: 0.5–1 for consensus) [50]; ii) the 70% cut-off for agreement and disagreement Likert-scales [51]; iii) the interquartile range (IQR) and Kendall’s W for the relevance and prioritisation of responses (IQR≤1 for consensus) [49].

The experts were identified using a snowball technique, based on: i) their professional expertise in one or more of the WEFE Nexus nodes; ii) their familiarity with the area of Apokoronas; iii) their involvement in the integrated management of natural resources [52]. In Delphi studies, samples are assessed based on the representativeness and expertise of the participants, rather than their numbers [43]. Participants were selected so that the group composition could reflect the diversity of knowledge stemming from different WEFE sectors and societal expressions. The total number of invited participants was 20, with five participants for each WEFE Nexus sector. Approximately one-third of those invited took part in the first round (n = 7). The absence of attrition rate between rounds suggests that the questionnaire was relevant to the experts and successful in terms of addressing the complexity of the WEFE Nexus thinking [53]. Regarding the optimal number of Delphi participants, there is enormous variability across studies [54]. Previous Delphi studies on the Nexus [21, 22] and natural resource management [46, 48] have used a similar number of participants. The chosen panel reflected the required balanced composition among WEFE sectors and was highly knowledgeable of the case study area. For a more detailed presentation of participants’ characteristics, please refer to S1 Table.

### 2.3 Focus groups methodology

Six focus groups were organised in the municipality of Apokoronas in Crete, which included thirty-nine participants from the tourism, food services, agricultural and processing sectors, as well as representatives of environmental groups and women’s collectives. The categories were defined based on the literature review and informal discussions. Each focus group comprised four to seven participants. Thirty-seven participants were of Greek ancestry, one was originally from a Balkan state and another from a country in Western Europe. All participants were born or had been living in Crete for over 30 years. The majority had completed their secondary education, attained their High-School diploma, or had higher education (university or technical training) degrees. Many participants held two jobs. For example, they might have owned a small business or worked seasonally in the tourist industry, and farmed their land during the winter months.

The focus group guide followed the structure described in Fig 2 and included questions that concern the interlinkages among the Nexus nodes in the region, and the conflicts between diverse local stakeholders and policy issues. The duration of the focus group discussions ranged from one hour to one hour and a half. The research team was composed of three researchers with extensive knowledge of the context in question. In most cases, the participants knew each other, and there was already trust and intimacy among them. The audiotaped focus group (FG) discussions were transcribed verbatim, cross-checked for accuracy and analysed using Thematic Analysis (TA) procedures, as outlined by Braun and Clarke [55]. The analysis proceeded through the following steps: familiarisation with the data, recognition of patterns across the data and the identification of themes. Excerpts from the focus groups are used in order to strengthen the line of argumentation.

### 2.4 Ethical approval and informed consent

The research was performed as part of the SIGMA-Nexus project, a research project funded under the European Commission H2020-PRIMA-2019 call (ID1943). The research project as a whole, including the FG and the DS, was approved by the Research Ethics Committee of the University of Crete (REC-UOC, A.P. 220/15.12.2020). The data for both FG and DS were collected during the summer months of 2021. For the Delphi survey, the online software Questback was used. The focus groups were held in person either in local restaurants or in a venue at the town hall of Apokoronas. In both cases, the participants were asked to read and sign an informed consent and respond to demographic questions.

## 3 Results

### 3.1 Delphi

#### 3.1.1 Sustainable development priorities

**Fig 3:**
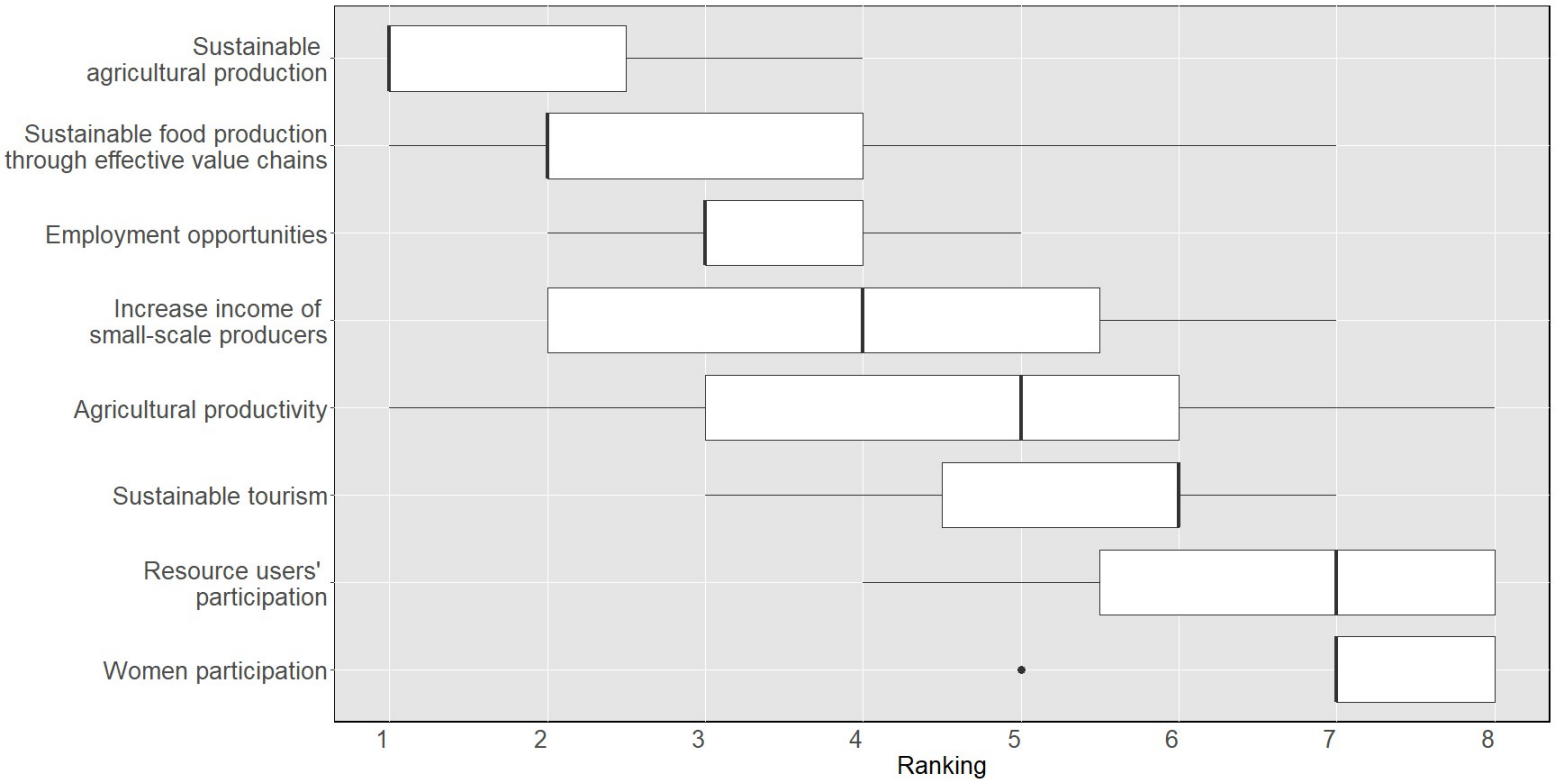
Prioritisation of socio-economic SDG targets for Apokoronas. KW after the first round was 0.53 (p < 0.001).

After responding to demographic and expertise-related questions in the first section of the survey, the experts were asked to rank in order of importance a list of socio-economic and environmental objectives (SDG targets) for the area of Apokoronas (Fig 2). The targets, strongly related to the WEFE Nexus domain, were adapted by Malagó et al. [31]. The idea behind this question is that interconnections among Nexus nodes cannot be identified without taking into consideration local development objectives and policy priorities [19]. The latter provides a benchmark for assessing the fit and relevance of specific Nexus practices. The SDG targets, in particular, were chosen because of their dual global and local relevance, and their connection with the WEFE Nexus [7]. As discussed in Section 2.2, the panel’s agreed ranking of priorities was assessed using Kendall’s W, a non-parametric statistic for assessing agreement among panellists [49]. While an overall consensus among experts was reached for the socio-economic objectives in round one, the consensus threshold for the environmental objectives was reached in the second round. As shown in Fig 3, in terms of socio-economic objectives, expert opinion gravitated towards the prioritisation of those targets related to sustainable agriculture and food production.

These two were ranked higher compared to sustainable tourism development, which was quite low on the list. Increasing the employment opportunities and income of small-scale producers were also ranked quite high among the proposed objectives for Apokoronas. This was reinforced by a panellist, who stressed the need to *‘enhance employment in the primary sector as a socio-economic solution for improving the management of resources in the area’*. This seems to be related to the fact that, in Crete, agriculture has become a part-time activity coexisting with seasonal work in the tourism industry. It has been observed that farmers in the area do not prefer farming methods that require full-time commitment or special management practices and, as such, they are subject to income diversification [56].

**Fig 4:**
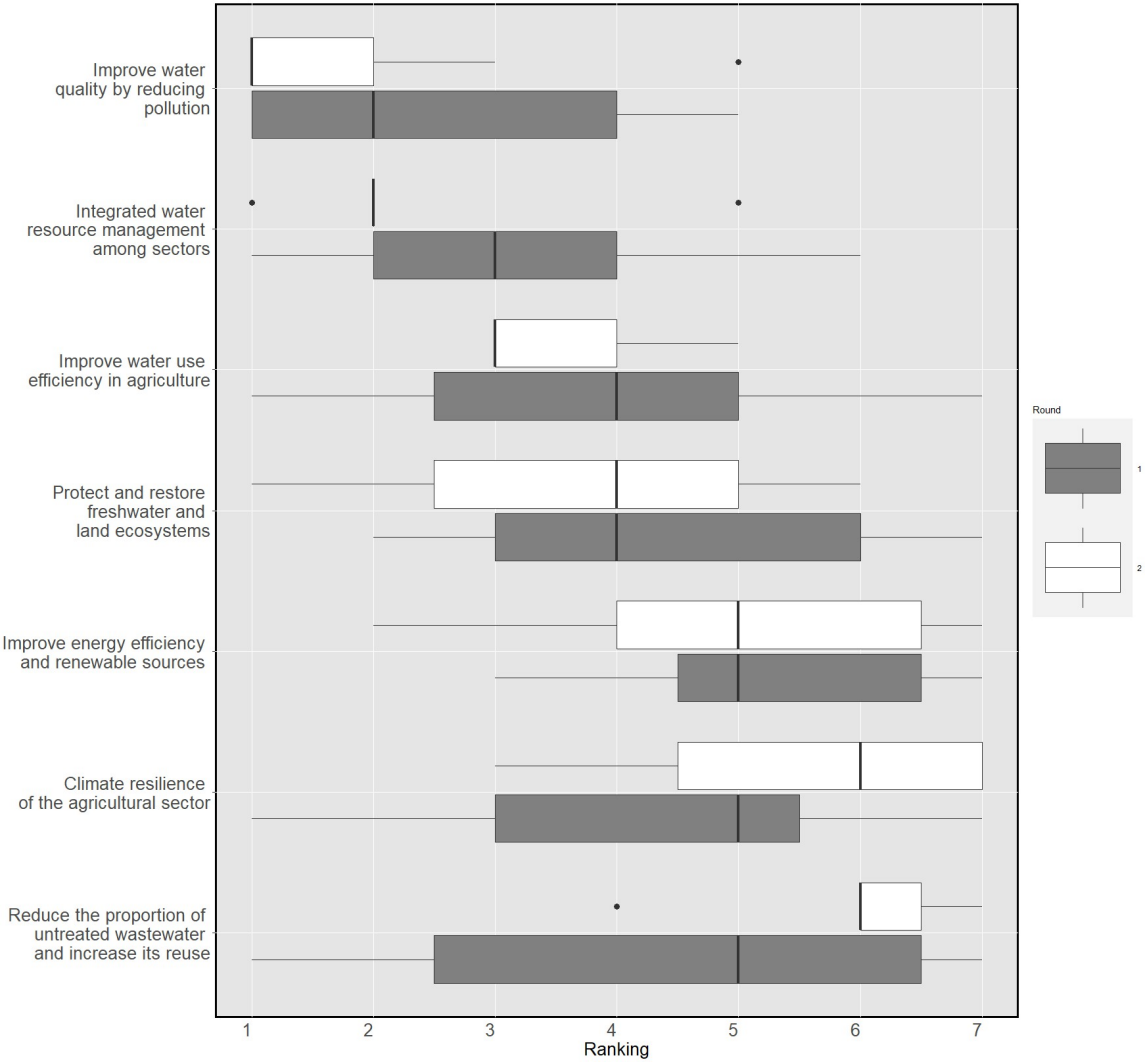
Prioritisation of environmental SDG targets for Apokoronas. KW after the second round was 0.54 (p < 0.001).

In terms of environmental objectives, all targets related to water security were considered a priority. Reduction of water pollution and integrated water resource management were prioritised, followed by the need to increase water use efficiency in agriculture. The role that water and land ecosystems play in the achievement of such goals was also recognised. Fig 4 shows how, between the first and second rounds, the objectives related to protecting the ecosystems and increasing climate resilience moved higher in the ranking, at the expense of those related to the sustainable management of wastewater and clean energy.

#### 3.1.2 Drivers and impacts

**Fig 5:**
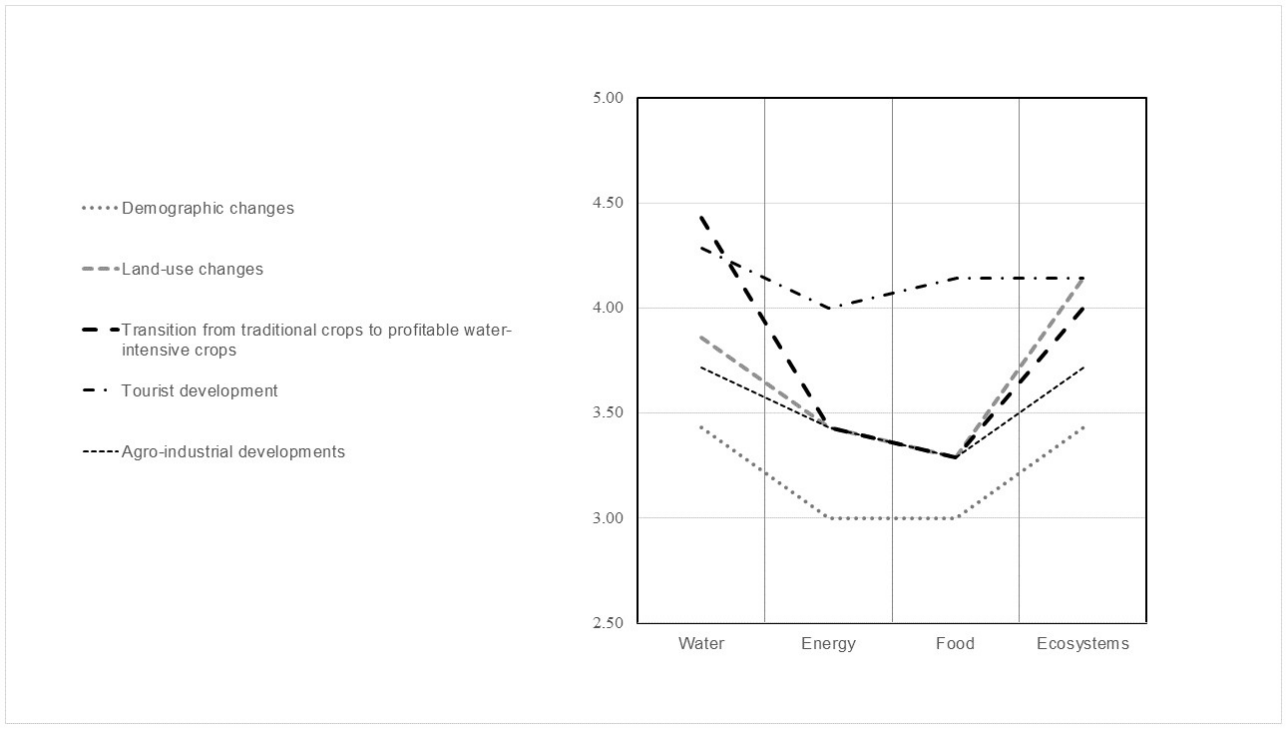
Impact of drivers of change on the WEFE sectors. Mean of the rating on a scale from 1 to 5, where 1 is "very low impact" and 5 is "very high impact". The coefficient of variation was below 0.3 for all ratings.

During the key informant interviews, the experts identified agriculture, tourism, land-use changes, urbanisation and transition to more water-intensive crops as the main drivers of change in Apokoronas. In the Delphi survey, the panel was asked to rate the impact of these five factors on the WEFE sectors. The experts agreed after the first round on the role that the different drivers play in the WEFE. As shown in Fig 5, according to the experts, tourism development and the transition to more water-intensive (and profitable) crops have the most impact on the WEFE Nexus in Apokoronas.

#### 3.1.3 Pressures

Thereafter, the experts were presented with a list of statements to identify the main pressures on the WEFE Nexus and their underlying driving forces. The experts were asked to indicate their level of agreement or disagreement with the proposed statements. The final list of pressures, following the second round of elicitation, is reported in Table 1. Surprisingly, only 29% (2 out of 7 experts) agreed on tourism development as a driver of pressure on WEFE resources, while opinions about the role of land management practices on the Nexus were mixed.

**Table 1:** Expert agreement on the pressures and causes of pressure on the WEFE Nexus in Apokoronas.

	Agree (%)	No opinion (%)	Disagree (%)	Consensus	Tot.
**Pressures**					
Increased tensions among water users regarding water allocation	83%	17%	0%	yes	6
Inadequate management of natural water resources leading to water stress	86%	0%	14%	yes	7
Over-exploitation of groundwater	71%	14%	14%	yes	7
Saltwater intrusion	71%	14%	14%	yes	7
Climate change impacts on the Nexus	71%	29%	0%	yes	7
Land-use changes and land degradation	86%	14%	0%	yes	7
Increased exposure to natural hazards (e.g., floods, fires, droughts)	100%	0%	0%	yes	7
Inadequate wastewater management	86%	0%	14%	yes	7
Tourism development	29%	71%	0%	yes	7
Negative impacts of farming on ecosystems	71%	14%	14%	yes	7
Water contamination from agricultural activities	71%	29%	0%	yes	7
Inadequate land management practices in agriculture	14%	71%	14%	yes	7
Climate change impacts on agricultural production	71%	0%	29%	yes	7
Transition to more water-intensive crops	86%	14%	0%	yes	7
Use of tourist sector resources is efficient	0%	29%	71%	yes	7
Use of farm resources is efficient	0%	86%	14%	yes	7
Energy use in agriculture is efficient	71%	0%	29%	yes	7
Organic farming is widespread	14%	57%	29%	no	7
**Causes of pressure**					
Current WEFE policies are integrated and account for sectoral impacts	29%	14%	57%	no	7
Current WEFE policies are up to date and take into account the impact of climate change	14%	14%	71%	yes	7
Coordination between WEFE authorities in Apokoronas is already in place and effective	0%	29%	71%	yes	7
Tourism development strategies in Apokoronas take into account the impact on WEFE systems	0%	14%	86%	yes	7
Water legislation and pricing mechanisms take into account water scarcity and externalities affecting other sectors	0%	29%	71%	yes	7
Lack of accurate and reliable data to support authorities’ decision-making	57%	43%	0%	no	7
Obsolete water network is the main cause of water use inefficiency	86%	0%	14%	yes	7
Lack of incentives to adopt innovations in agriculture	57%	29%	14%	no	7
Farmers are resistant to innovations	0%	43%	57%	no	7
Lack of incentives to adopt sustainable soil management practices	86%	0%	14%	yes	7
Lack of adequate knowledge on how to apply sustainable agricultural practices	71%	29%	0%	yes	7
Farmers participating in water users’ associations or farmers’ organisations are more efficient	57%	14%	29%	no	7
Farmers participating in water users’ associations or farmers’ organisations are more likely to apply sustainability practices	71%	29%	0%	yes	7
Larger farms are more efficient in resource use compared to small ones	43%	29%	29%	no	7

The experts have confirmed the presence of increased tension among different water uses (e.g., domestic, tourism and agriculture). They have also confirmed that water stress mainly stems from the inadequate management of water resources, rather than being the result of water scarcity. This corroborates a previous observation by Chartzoulakis et al. [57] and Tzanakakis et al. [34].

Further, the role of tourism on the WEFE in Apokoronas was viewed with a high degree of uncertainty. The plurality of opinions expressed can be related to the fact that none of the experts specialised in tourism, but also to the widespread recognition of tourism as an instrument to boost ecosystem preservation, especially in the case of Lake Kournas. This ambiguity was articulated by one expert in the following terms: *’the tourist development of the lake will contribute to its preservation*, *but not to its upgrading’*. In addition, opinions about climate change, already seen to affect the WEFE resource base and, in particular, agricultural production, were also mixed.

Regarding energy, the key informant interviews pointed out the existence of a link between water and energy production. The panel confirmed the synergy between the two sectors, as well as the potential for this relationship to be exploited in the near future. Nevertheless, some experts also highlighted how rising energy prices limit the development of agricultural holdings, arguing for the promotion of desalination units and, hence, alternative water sources. The relationship between the two sectors is, thus, not seen in terms of trade-offs, but rather in terms of synergies. Despite the fact that the energy sector as a whole does not seem to be highly impacted by the rest of the sectors and is certainly not viewed as an issue of concern for the area by the experts, a plurality of opinions exists about the level of energy use efficiency in agriculture. Another point of disagreement can be found in relation to the adoption of sustainable farming practices, and in particular organic farming. One expert commented on the *’negative impact of agriculture on the ecosystems’* with more sustainable farming practices such as ’organic agriculture’ failing to scale up because of the absence of *’the expected economic results’* and *’environmental consciousness for the majority of farmers’*.

Table 1 reports the underlying causes of pressure on the WEFE Nexus, as identified by the experts. Among 14 proposed statements, seven reached relevant statistical consensus (APMO above 70%). The threshold was not reached in those questions describing the role of farming activities in the WEFE Nexus, most probably because a higher level of knowledge in agriculture was required. Nevertheless, after the second round, the experts converged to the most popular opinions in most cases. The panel did not reach a definitive consensus on whether current water, agricultural, energy and environmental policies are sufficiently integrated taking into account impacts to other sectors. By contrast, they agreed that the existing instruments do not adequately address the impact of climate change on WEFE systems. The panel also argued that there is a lack of coordination among WEFE authorities affecting resource management in the area. In addition, they agreed on the following underlying causes of pressure: local tourism development strategies do not take into account the impact of tourism operations on WEFE systems to any satisfactory degree; the water network is near-obsolete, water losses and reckless use of water are among the main reasons for water use inefficiency; lack of accurate and reliable data, and cumbersome water legislation and pricing mechanisms.

#### 3.1.4 Responses

In the final section of the survey, a list of 24 suggestions for increasing WEFE Nexus integration and addressing the challenges previously identified in the survey was proposed to the panellists. The experts were asked to rate the relevance of two groups of actions: (a) governance and policy-related responses, and (b) socio-economic and technical responses. In terms of consensus, low heterogeneity was found among expert opinions. In the first round, only six out of twenty-four responses showed an IQR value greater than or equal to 1. After the second round, the experts converged towards the majority opinion and the six innovations registered a reduction in their IQR range. This means that all responses were considered to have certain relevance by the experts.

**Fig 6:**
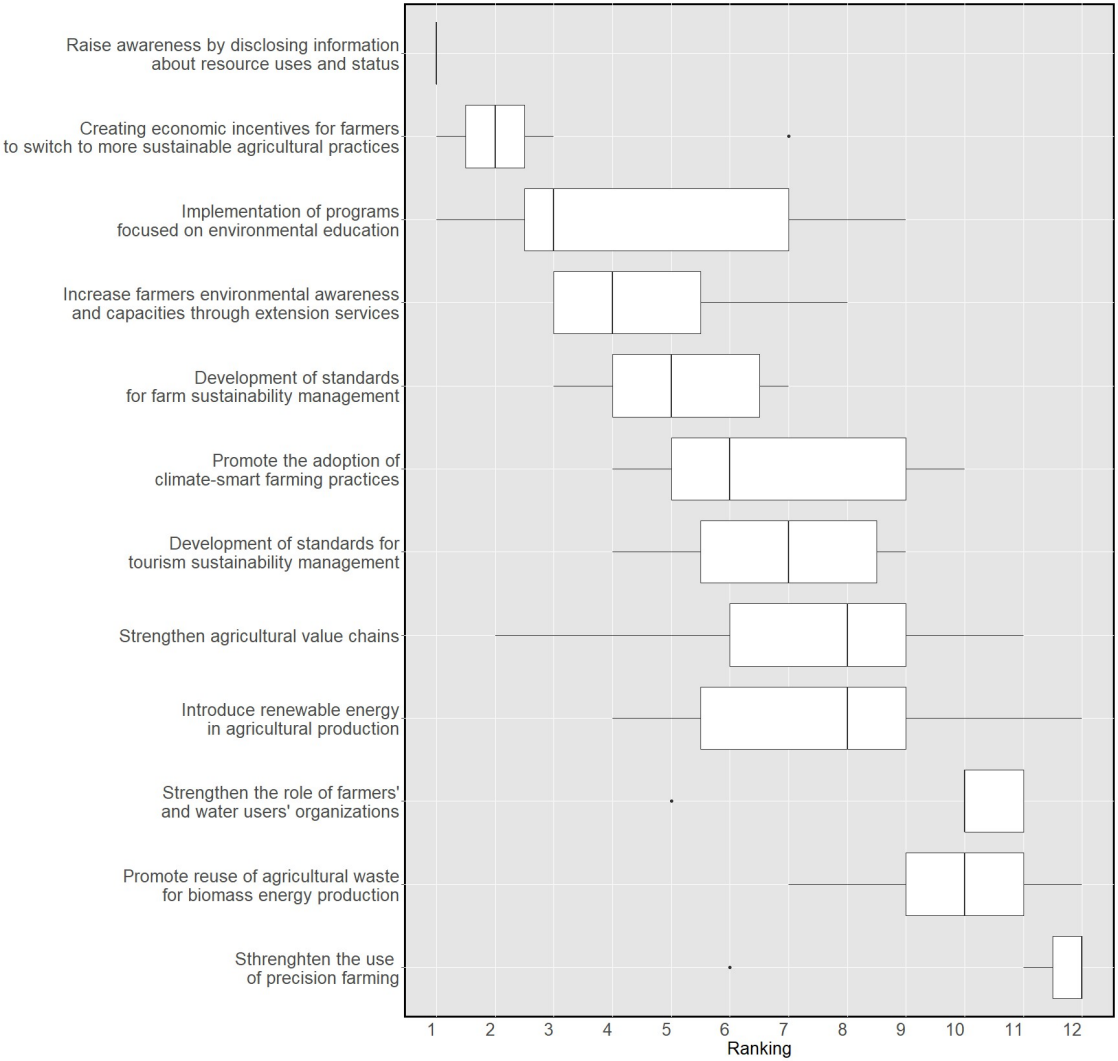
Prioritisation of governance and policy responses. Experts’ ranking on a scale from 1 to 12 (KW: 0.43).

**Fig 7:**
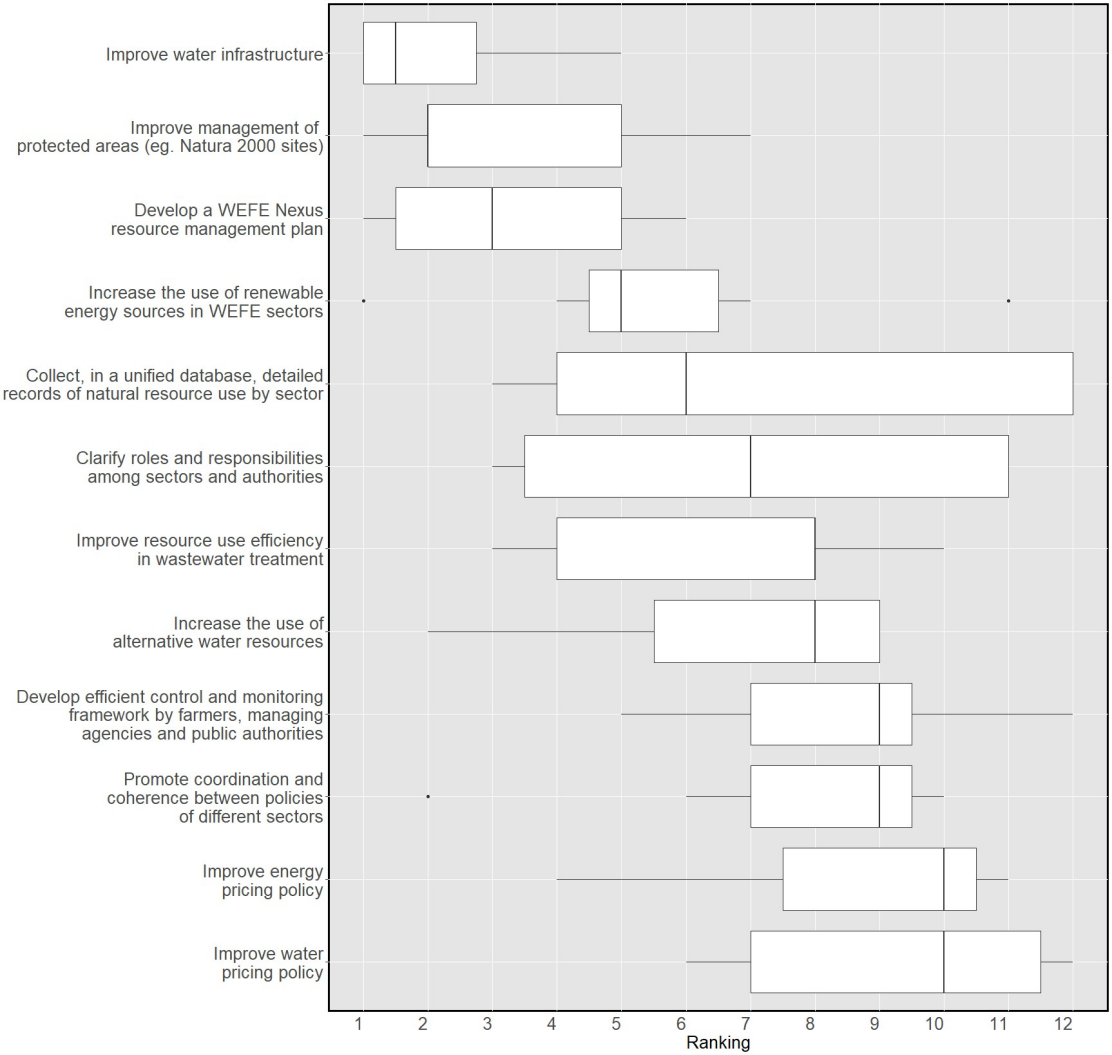
Prioritisation of socio-economic and technical responses. Experts’ ranking on a scale from 1 to 12 (KW: 0.62).

In the second round, the panel was asked to rank on a scale of 1 to 12, in order of importance, the responses in the two groups. Considering the low heterogeneity of opinions observed in the first round, this ranking aimed to test, using Kendall’s W, the experts’ agreement on their prioritisation. The final ranking is reported in Figs 6 and 7. For the governance and policy-related responses, a Kendall’s W of 0.43 was obtained. This indicates that despite all suggestions being relevant, a low-moderate level of agreement exists on their prioritisation for WEFE integrated resource management. This disagreement is statistically significant. A higher agreement level was found in the ranking of the socio-economic and technical innovations. The ratings showed a statistically significant Kendall’s W of 0.62.

Overall, a number of responses aimed at improving resource management was rated as extremely relevant, such as: i) upgrading water infrastructure; ii) enhancing the management of protected areas (e.g. Natura 2000 sites); iii) developing a WEFE Nexus resource management plan; iv) collecting, in a consolidated database, detailed records of natural resource use by sector; and v) increasing the use of renewable energy sources in the water, food and tourism sectors. Raising awareness by disclosing information about resource use and status was also considered of extreme relevance, followed by the need to create economic incentives for farmers to switch to more sustainable agricultural practices, and the need to implement programmes focusing on environmental education. This is in line with a comment by an expert arguing that ’*the beliefs of the local population on the state of natural resources do not align with the effective conditions of the Nexus resource base*’. Equally interesting is the importance given to the efficient management of protected areas, referring in particular to the Natura 2000 site of Lake Kournas. This suggests that, according to the panel, conservation efforts are relevant for the area. Similarly, the experts highlighted the importance of developing sustainability standards on the part of the farming and tourism sectors.

### 3.2 Focus groups

**Fig 8:**
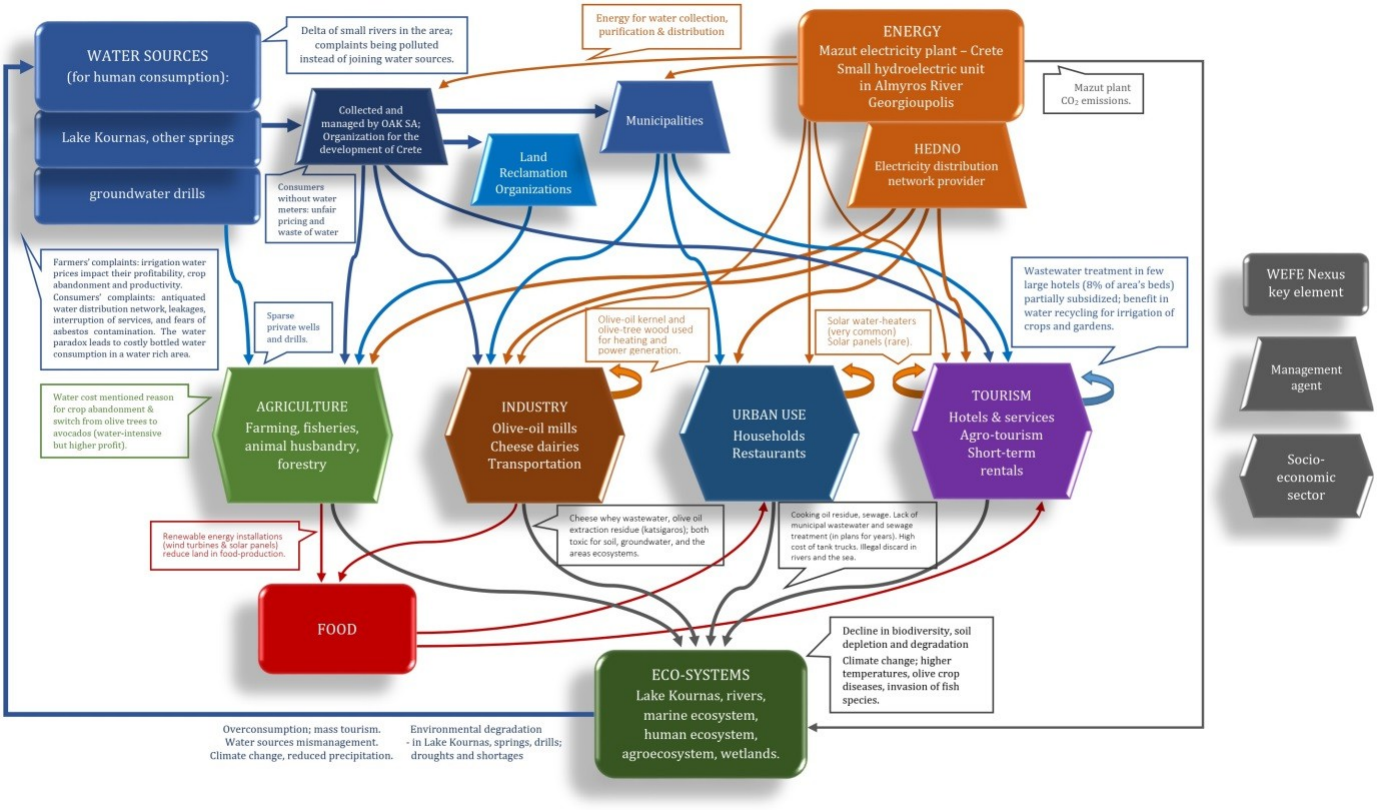
WEFE Nexus interconnections in Apokoronas.

The analyses from the FG discussions in the Apokoronas area revealed the following thematic units: i) WEFE Nexus understandings, nature’s endowments and "the water paradox"; ii) Sustainability, existing policies, proposed amendments and enforcement obstacles. Fig 8 visually depicts the first thematic entity and the interconnections among the WEFE Nexus nodes resulting from the focus group analysis, while Table 2 presents the second thematic entity, namely the policy recommendations and solutions to the natural resource sustainability challenges identified by the participants.

**Table 2:** FGs recommendations for addressing natural resources sustainability problems in Apokoronas.

Sector(s)	Recommendation(s)
**Food**	1. Empowerment of agricultural directorates, agronomists and farming cooperatives
**Water—Ecosystems**	2. Transparency in water management and in WEFE governance, and the establishment of one municipal organisation that is adequately staffed
**Ecosystems**	3. The construction of a municipal WWTP; a key step for the sustainability of the region
**Food—Water**	4. Link local agricultural and livestock production to the local tourism market, and develop and apply an equitable water pricing system
**Tourism**	5. Promotion of alternative forms of tourism (ecotourism and agrotourism) and reduction of mass- and over-tourism
**Tourism—Ecosystems**	6. Zoning policies that maintain a balance among tourism, urban expansion and environmental sustainability are required
**Food—Ecosystems**	7 Implementing policies that can bolster an organic farming support scheme as a key driver of dynamic agricultural growth, while ensuring the sustainable functioning of ecosystems and food safety

#### 3.2.1 WEFE Nexus understandings, nature’s endowments and "the water paradox"

Participants provided multiple examples regarding the connections between different nodes of the WEFE Nexus. For example, some of the farmers from the Armenoi village are forced to use brackish water from the Zourbos springs in order to irrigate their avocados, which is considered to affect production. In another example, as an olive-producing municipality, Apokoronas’ many oil producers described how the biofuel produced by processing olive kernels is used to generate energy for heating their homes or running their businesses. Olive kernel biofuel is also used to produce energy to pump water from wells. In terms of economic profitability, the transition to more profitable but also more water-intensive crops (mainly avocado) is increasing concerns regarding the impact of these practices on the Water-Ecosystems-Food sectors of the Nexus. Moreover, despite being a predominantly olive- and dairy-producing municipality, proper management of olive mill wastewater (OMWW) and cheese whey wastewater (CWW) is inconsistent and not regularly enforced in Apokoronas. Focus group discussions veered to the impact of the olive mill by-product, *Katsigaros*. Participants described how its runoff into aquifers and soils leads to local ecosystem degradation. Along the same lines, participants mentioned how dumping cheese whey wastewater (CWW) is another common practice in the region that adversely affects the local ecosystems.

From the multitude of examples presented in Fig 8, this analysis will elaborate on three that dominated the focus group discussions: the overuse of plastic bottles, wastewater treatment and renewable energy. The first example concerns the impact of the overuse of bottled water on WEFE nodes. In all the FGs, participants described Apokoronas as an area that is blessed, endowed and privileged by nature, with ample water sources. They expressed their opinion on all the nodes of the Nexus, but discussions were prominently water-centred since water issues are vital for the area. Nevertheless, participants described the fact that their land is ’rich in water’ and endowed by nature, yet faces a multitude of problems related to water management, as ’a paradox’.

Overuse of plastic bottles is the result of the poor quality of tap water, forcing participants to resort to bottled water. There was a sense of injustice that permeated these discussions in the FG, particularly since an award-winning brand of water is bottled and distributed to the rest of Greece and abroad from springs in the area (Lefka Ori, the village of Stylos). Their biggest concern, however, was the deterioration in water quality and water resources, rendering tap water unusable and leading participants to buy expensive bottled water in bulk. As proper recycling policies and the corresponding mentality are lacking, participants described how dependency on bottled water leads to plastic inundation. Plastic gets dumped into the water sources and affects marine, lake and river ecosystems, while microplastics are entering the food chain, accumulating in the bodies of birds and other animals.

Regarding wastewater, participants stated that a wastewater treatment plant (WWTP) had been in the making for years. As many local businesses dump their sewage directly in the water sources and in the natural environment, many nodes of the WEFE Nexus are adversely impacted. This practice has immense negative effects on marine habitats, food quality and food production, as well as environmental sustainability. Concerning wastewater treatment, many participants believed that big businesses had significant advantages over smaller family-owned enterprises. This is a serious point of contention because it affects households and family-run businesses in the entire area in question.

According to the FG participants from the tourism sector, large hotels are partially exempt because many of them have the capacity to process sewage for at least some of their units. Smaller hotels and businesses do not have sewage processing capabilities and shoulder hefty costs for transporting water sewage to another municipality’s sewage processing plant.

In general, there was homogeneity of opinions in the discussions, but there was disagreement over whether small businesses could adopt such sustainable practices. Vassilis, Stelios and Giorgos, whose real names are not revealed for confidentiality purposes, from the tourism focus group vehemently disagreed:

***Vassilis***: *You are obliged to do something about sustainable practices*.

***Stelios***: *It’s not your obligation*, *it’s the obligation of the municipality*. *The municipality is the starting point […] it is the base*. *For me*, *the problem starts from your base*. *If your base does not have a wastewater treatment plant*, *they can’t come to me and say*, *’why do you dump your sewage*?*’*.

***Giorgos***: *Since you are obliged to have your own wastewater treatment plant*, *I can’t understand you*.

***Stelios***: *Since we have reached this impasse*…

***Vassilis***: *And what can one do with your wastewater*?

***Giorgos***: *And how are you going to function*? *Don’t start your business*? *If you don’t you confront a more complex predicament*.

Vassilis and Giorgos are representatives of large resort hotels, which have facilities such as solar photovoltaic panels and sewage/wastewater treatment plants. They also reuse water to grow some crops that are then used in their kitchens. They explained that not all wastewater is processed due to the limited capacity of the sewage treatment systems they have. In this dialogue, they both claimed that wastewater treatments plants are primarily privately owned facilities and should be ’obligatory for all’ businesses. They both pressed Stelios by persistently interrupting him and posing questions about his responsibilities. Stelios, the owner of a smaller business, strongly disagreed and argued that establishing such practices is not financially possible for his business, and that the management of wastewater should be the responsibility of the municipality. This verbal encounter reflects wider societal conflicts regarding power inequalities, state responsibilities and the capacity of smaller tourist establishments to adopt sustainable practices.

The differences of opinion also centred on renewable energy. Wind energy was a point of contention because many participants believe that wind turbines will lead to land fragmentation (construction or roads in mountainous and forest landscapes), change of land use at the expense of the ecosystems, and are noisy and aesthetically unappealing on mountain peaks. Other participants focused on the low operating costs of wind farms, Crete’s “Meltemi winds” and the White Mountain range in the case study area that forms a natural barrier for winds. Solar energy via solar-PV installations also drew differences in opinion with some participants expressing beliefs that such installations compete with farmland, “take up lots of land” and the panels’ efficiency levels are relatively low and are hampered by storage difficulties.

In the participants’ examples of sustainable (e.g., wastewater recycling) and unsustainable (e.g., the preponderance of bottled water use) practices, the interconnections between the WEFE Nexus nodes were highlighted. The majority of the examples that participants offered focused on the interconnections between the water and food nodes and their crucial role in farming. Despite the fact that many focus group questions pertained to the significance of energy and ecosystems, participants were less reflexive about these two nodes and their impact on their livelihoods. Moreover, participants were hesitant in delving into the complicated relationship between tourism’s contributions to economic growth and its negative environmental and sustainable use of natural resources.

#### 3.2.2 Sustainability, existing policies, proposed amendments and enforcement obstacles

This theme delineates participants’ policy proposals regarding the sustainable use and management of natural resources. Although participants valued Apokoronas’ natural water capital and its diverse ecosystems, they elaborated on their concerns regarding relevant policies and their enforcement. In this theme, the focus is on three vital policy issues: i) the effects of over-tourism; ii) the pricing of water and the future of local agriculture and farming; and iii) the transparency of natural resources management.

The expansion of the ’tourism industry’, according to the residents who live and work in the area, has created more jobs, economic growth, and possibilities for the advancement of people’s well-being. Nevertheless, they believe that this industry contributes disproportionately to natural resource depletion in all the nodes of the WEFE Nexus and, according to some, these deleterious effects are currently evident in the area. Participants discussed the problems involved in resource sustainability, and described the current state of affairs as a voracious consumption of resources. The participants from the water collective focus group noted that most water bodies, including estuaries, the coastal zone and even the sea, have been degraded mainly to assuage short-term economic gains for a few people. As a result, not much attention is paid to the long-term economic and environmental sustainability of the study area. In two discussions regarding "over-tourism", participants proposed "ecotourism", "agro-tourism", and other more environmentally friendly forms of tourism as solutions for the reduction of trade-offs among sectors.

Local farmers noted that they struggle to compete with large multi-national commodity markets and are under pressure from tourism expansion, facing serious challenges to secure public support. They consider their future to be unviable and unsustainable. As shown in Table 2, one proposed solution is the development and implementation of policies empowering agricultural directorates, agronomists and farming cooperatives. This includes creating a link between local agricultural and livestock production and the local tourism market. Participants noted that the tourist industry has eclipsed their contribution to Apokoronas’ economy. They discussed the fraying of bonds in their rural communities, and disclosed pressures to reinvent farming in a way that balances ecological, social and economic concerns. Farmers outlined their need for programmes that provide support, guidance and know-how about crops’ water needs, and adaptation to climate change, including biological pest control.

Regarding water management and distribution conflicts, participants proposed transparency and alignment with governmental authorities and an equitable water pricing system. They advocate for a water pricing system that is fair for farmers, household consumers, food and processing businesses and the tourist industry. They enumerated many problems, including over-exploitation of water sources during the summer months, low water efficiency (in farming, businesses and homes), absence of a quality monitoring system, lack of information regarding water quality (publication of water analyses data), an outdated and faulty distribution system with frequent leaks and asbestos pipes, salt-water intrusion, inadequate cooperation amongst the different agencies that are responsible for water, and lack of a modern recycling and reuse system.

One of the six focus groups comprised members of a local water collective. In the following dialogue between two members of the water collective, this sense of vagueness regarding the management of natural resources is brought to the foreground:

***Alexia***: *Have we discussed the asbestos pipes*?

***Giannis***: *Yes*, *there are multiple problems*, *the asbestos pipes*, *which* […] *people do not readily acknowledge in our region* [the asbestos pipes] *are some kilometres*, *we do not exactly have data*, *we do not exactly have data*.

***Alexia***: *It’s all very unclear*, *this is the problem*.

***Giannis***: *Yes*, *that there is misinformation*, *data concealment*, *I do not know exactly what’s happening*. *The truth is that I can’t say something with absolute certainty*.

Members of this group, which operates through open assemblies and participatory processes, expressed their ambivalence, bewilderment and uncertainty towards the existence of old water lines made of asbestos, a material that has been phased out. Giannis replied very hesitantly to Alexia’s request to discuss the topic and explained that, although they are informally conducting their own research, they cannot provide clear evidence. This was a dominant interaction pattern in this focus group, where one participant would disclose a piece of information, but would immediately be challenged by other participants on the reliability of their assertion. The members of the group stated that they are considering ways to demand and gain access to water quality data, arguing that water testing results from certified laboratories are not posted publicly. In this respect, they were vocal about their belief that being informed about the quality of water is a democratic right of all citizens.

Policies in the energy sector and land use were also deemed urgent. The participants stated that policies in the energy sector should include the regulation of energy prices and the provision of viable programming by amending the “Save-Spare” initiative from “energy inefficient buildings” to nearly zero-energy buildings. Energy needs should be met from renewable sources, including those produced on-site or locally. As the participants explained, that different “renewable energies that are storable” and that “replace Crete’s reliance on the fossil fuel mazut” are necessary. The focus group discussion on land use brought up the necessity to change zoning policies that were not fully applied and planning laws that are incomplete. The existing zoning policies are “*sporadically and unfairly enforced leading to the construction of tourist facilities on the coastline*”.

## 4 Discussion

The use of multiple research methodologies is common practice in qualitative research. The triangulation of results from different methods and data sources allows for a more comprehensive understanding of the study object, provides more robust confirmation of findings and includes diverse perspectives [32]. The use of DS and FGs in this study aimed specifically to explore whether the combination of these methods could capture more holistically the WEFE Nexus interrelations and the perspectives of different stakeholders. As the result of quantification, biophysical flows among Nexus nodes cannot be easily contested; the management of resources, however, is determined by socio-economic needs, and cultural and historical forces that influence subjective perceptions of interconnectedness, and present challenges and opportunities for their sustainable use.

This study applied two different methodologies bringing together two distinct dimensions of the Nexus approach. On the one hand, the study accounted for the technical and managerial dimensions of the Nexus thinking by asking WEFE experts about the main challenges to sustainable resource use, and relevant solutions in light of local development priorities. On the other hand, the study attempted to capture the perceptions and experiences of local populations regarding the use and management of natural resources, including their views on justice and power inequalities. The integration of these two perspectives may lead to more informed WEFE Nexus policy initiatives, which take into account the voices and perspectives of diverse local stakeholders.

The relevance of both methods was considerable to all three research questions. Regarding the first question about how diverse stakeholders understand and frame the interconnections and interdependencies across the WEFE sectors, the comparison revealed multiple perspectives with different points of convergence. Both groups (Delphi panellists and Focus Group participants) confirmed that Nexus thinking in Apokoronas has a strong focus on water. Bridging the gap between water availability and demand and improving water quality were central concerns. The focus groups participants underlined the power inequalities and injustices and highlighted implications for the rest of the Nexus nodes (e.g. overuse of plastic bottles affecting the ecosystems and the negative impact of the use of brackish water for crop production). The Delphi captured the underlying causes of the "*water paradox*" pervading the WEFE Nexus, which include, among others, the low-level coordination between water and WEFE authorities, the fast-becoming obsolete water network, and inadequate water legislation and pricing mechanisms. Nonetheless, while the need for improving water pricing policies was firmly pointed out by the focus groups participants, who advocated for a more equitable water pricing system, local experts did not consider this a priority. This is probably due to the experts’ concerns about the different levels of jurisdictions that need to come together in order to effect an overhaul of the pricing system.

Concerning the second research question, on how participants suggested improving the WEFE Nexus, several opportunities for a more integrated WEFE resource management in Apokoronas were found. The groups converged in the main ways to address the major Nexus challenges and resource use conflicts. Both groups acknowledged the importance of environmental education and the proper disclosure of information. The latter was raised by the experts, who called for the public availability of all relevant data for decision-making. Focus group participants attributed a more political meaning to information dissemination, referring to the lack of transparency in the use of natural resources. While the Delphi experts identified areas of macrointervention at the management and policy levels (such as improving the management of protected areas or creating a WEFE Nexus management plan), FG participants reflected on specific steps towards change, such as the strengthening of agronomic services, zoning policies and local value chains. A tendency to prefer top-down interventions to bottom-up grassroots movements can be observed in both groups, suggesting that the reduction of complexity and the integrated management of resources are considered the responsibility of policymakers.

Finally, for the third research question on the potential of qualitative methodologies in Nexus designs, elicitation and reflexive thinking inherent in both methods contributed to identifying node interlinkages and resource management challenges for Apokoronas. FG participants could break down complex interactions even if not conceptualising them as a Nexus. The discussions also allowed the detection of already applied Nexus practices in the area. These include, for instance, the production of biofuel as a by-product of olive farming or the reuse of water from hotel wastewater treatment systems. Despite the clarity with which focus group participants showcased the interlinkages of the four nodes in their area, they concentrated mainly on those resource management problems that had a direct impact on their livelihood (e.g., municipal wastewater sewage treatment), while disregarding other ecosystemic challenges (e.g., biodiversity loss) that are not directly relevant to human well-being. The Delphi approach filled this gap, with experts outlining the role of different factors (drivers, pressures, causes) in determining Nexus sustainability as a whole. Delphi experts focused on challenges at the municipality level, but interpreted them in the regional context. They provided an overall interpretation of the variables that need to be considered when conducting WEFE Nexus assessments and, in that way, a sense of the relative importance of each issue was obtained.

On these grounds, it is possible to contend that the valuable insights of both FG participants and Delphi experts were complementary to one another. On a micro-level, FG discussions centred on the economic concerns of Apokoronas’ residents, regarding how the intricate connections between the decline of agricultural production, the outdated zoning policies and over-tourism pervaded the use of natural resources in the area. On a macro-level, the Delphi experts recognised the primary importance of the protection and restoration of ecosystems in the area, as a means to manage sustainably the rest of the nodes in the nexus. Further, by triangulating the results from the two methods, contrasting viewpoints about the impact of certain activities were identified. An example is the case of the tourism sector, which, although not explicitly considered in the WEFE Nexus, dominated the discussions. Delphi experts considered tourism as the sector with the most impact on the four WEFE nodes. Nonetheless, they did not prioritise sustainable tourism over the strengthening of sustainable agricultural production. Such an ambivalent position regarding tourism expansion was also observed among FG participants, who acknowledged that tourism was an important economic sector and, at the same time, the driver behind the depletion of natural resources in the area. To the contrary, agriculture, a prominent marker of local identity, was seen as retreating in the face of tourism as an economic activity. Lack of adequate knowledge and incentives to adopt improved management practices seems to be at the heart of the WEFE unsustainable farming systems and agro-industrial activities. The absence of expected economic benefits and low environmental consciousness reduces the proclivity among farmers to adopt environmentally friendly innovations. While Delphi captured the environmental and social dimensions of farming sustainability, the indignation of farmers who struggled to make ends meet was evident in the FGs.

This study added useful insights regarding the socio-economic and environmental trade-offs between WEFE sectors. Echoing a point made by several scholars [13, 14, 58], it is argued that neglecting the role of social, political and economic forces and local identities in the interlinkages between natural resources and decision-making can be detrimental. A remarkable study in this respect, which accounted for the context-specific, historical perspectives of people in Crete, is the study of Siamanta and Dunlap [58]. The authors investigated wind energy development in Crete and argued that local opposition to wind parks is deeply associated with the population’s past struggles against foreign powers. Following Siamanta and Dunlap [58] and ’*in contrast to the rather depoliticised and ahistorical treatment of social order and context in the dominant energy-water-food Nexus literature*’ [22], this study explored qualitative approaches to situate the participants’ experiences within their proper social, political and economic contexts. Therefore, the argument proposed in this study is that the WEFE Nexus is not a static, abstract, fixed concept, but rather a flexible, dynamic and empirically grounded construct that accounts for local particularities in the use of natural resources.

## 5 Conclusion

One of the challenges of the Nexus framework is how to bridge the technical and managerial dimensions of the WEFE interconnections, and the priorities and perceptions of local resource users and stakeholders involved in resource management. Since the introduction of the concept, approximately one-quarter of Nexus studies have been based on qualitative methods [15]. This study moved in the same direction by applying novel combinations of qualitative methodologies in Nexus research designs. Two methods, Delphi and Focus Groups, have been combined to investigate the WEFE Nexus in Apokoronas (Crete) and explored the added potential of this approach.

Each with its strengths and weaknesses, the two methods have proven complementary to each other. The Delphi was designed to overcome silo approaches in Nexus thinking by eschewing confrontation among WEFE sectoral experts. FGs seek participant interaction to stimulate conversation that would lead to the identification of common concerns and the sharing of opinions. Given the complexity of the Nexus discourse, the risk of unbalanced assessments among nodes, and the technical and societal dimensions pervading the different sectors, the combination of these two techniques provided grounds for a more sober analysis. For instance, FG discussions capture motives and perspectives that cannot be observed in an expert-led Delphi survey. The integration of the two methods is particularly suited for local WEFE Nexus assessments seeking to collect information for policy strategies aimed at improving Nexus resource management. The study identified reciprocal causal relationships among Nexus systems, which offer valuable insights for policy interventions and, from a research perspective, can be used to inform the development of further empirical studies using a Nexus approach. It also revealed nuances and different perspectives even in the face of low heterogeneity between and within groups. Based on the above, it is possible to conclude that such a combination of methods can provide useful insights on sectoral and societal conditions even in heavily diversified contexts.

## Supporting information

S1 Table Delphi study: Socio-demographic characteristics of participants. (DOCX)
