# Supplementary material for: Implementing a combined Delphi and Focus Group qualitative methodology in Nexus research designs—The case of the WEFE Nexus in Apokoronas, Crete
Source: PLoS One. 2022 Jul 14;17(7):e0271443. doi: 10.1371/journal.pone.0271443 (PMC9282543; doi:10.1371/journal.pone.0271443)
Supplement: S1 Table — (DOCX) [file pone.0271443.s001.docx]

| **S1 Table. Delphi study: Socio-demographic characteristics of participants** | | |
| --- | --- | --- |
|  | N of experts | |
| **Gender** |  | |
| Male | 5 | |
| Female | 2 | |
| **Age** |  | |
| <41 | 0 | |
| 41–50 | 3 | |
| >51 | 4 | |
| **Organisation** |  | |
| University or research organisation | 3 | |
| International organisation | 1 | |
| Water organisation (e.g., water cooperative) | 2 | |
| Self-employed | 1 | |
| Other | 0 | |
| **Years of professional experience** |  | |
| 10–19 | 3 | |
| 20–29 | 3 | |
| > 30 | 1 | |
| Share of experts with professional knowledge in specific WEFE sectors | | |
|  | Low | Medium–High |
| Water | 43% | 57% |
| Energy | 43% | 57% |
| Food | 29% | 71% |
| Ecosystem | 14% | 86% |
| Share of experts with professional knowledge in WEFE Nexus and natural resource management | | |
|  | Low | Medium–High |
| WEFE Nexus | 43% | 57% |
| Resource management | 14% | 86% |
